# Identification of Tumor Endothelial Cells with High Aldehyde Dehydrogenase Activity and a Highly Angiogenic Phenotype

**DOI:** 10.1371/journal.pone.0113910

**Published:** 2014-12-01

**Authors:** Hitomi Ohmura-Kakutani, Kosuke Akiyama, Nako Maishi, Noritaka Ohga, Yasuhiro Hida, Taisuke Kawamoto, Junichiro Iida, Masanobu Shindoh, Kunihiko Tsuchiya, Nobuo Shinohara, Kyoko Hida

**Affiliations:** 1 Department of Vascular Biology, Hokkaido University Graduate School of Dental Medicine, Sapporo, Japan; 2 Division of Vascular Biology, Institute for Genetic Medicine, Hokkaido University, Sapporo, Japan; 3 Department of Cardiovascular and Thoracic Surgery, Hokkaido University Graduate School of Medicine, Sapporo, Japan; 4 Department of Orthodontics, Hokkaido University Graduate School of Dental Medicine, Sapporo, Japan; 5 Department of Oral Pathology and Biology, Hokkaido University Graduate School of Dental Medicine, Sapporo, Japan; 6 Department of Renal and Genitourinary Surgery, Graduate School of Medicine, Sapporo, Japan; Osaka University Graduate School of Medicine, Japan

## Abstract

Tumor blood vessels play an important role in tumor progression and metastasis. It has been reported that tumor endothelial cells (TECs) exhibit highly angiogenic phenotypes compared with those of normal endothelial cells (NECs). TECs show higher proliferative and migratory abilities than those NECs, together with upregulation of vascular endothelial growth factor (VEGF) and VEGF receptor 2 (VEGFR2). Furthermore, compared with NECs, stem cell markers such as Sca-1, CD90, and multidrug resistance 1 are upregulated in TECs, suggesting that stem-like cells exist in tumor blood vessels. In this study, to reveal the biological role of stem-like TECs, we analyzed expression of the stem cell marker aldehyde dehydrogenase (ALDH) in TECs and characterized ALDH^high^ TECs. TECs and NECs were isolated from melanoma-xenografted nude mice and normal dermis, respectively. ALDH mRNA expression and activity were higher in TECs than those in NECs. Next, ALDH^high/low^ TECs were isolated by fluorescence-activated cell sorting to compare their characteristics. Compared with ALDH^low^ TECs, ALDH^high^ TECs formed more tubes on Matrigel-coated plates and sustained the tubular networks longer. Furthermore, VEGFR2 expression was higher in ALDH^high^ TECs than that in ALDH^low^ TECs. In addition, ALDH was expressed in the tumor blood vessels of in vivo mouse models of melanoma and oral carcinoma, but not in normal blood vessels. These findings indicate that ALDH^high^ TECs exhibit an angiogenic phenotype. Stem-like TECs may have an essential role in tumor angiogenesis.

## Introduction

Tumor angiogenesis is essential for tumor growth and metastasis, and plays an important role in cancer progression [1]; therefore, inhibition of tumor angiogenesis is a valuable approach for cancer therapy [2]. Although anti-angiogenic therapy prolongs the survival of patients with certain types of cancer, less responsiveness and side effects have been reported in patients with some types of tumors [3]. Recently, it has been revealed that tumor endothelial cells (TECs) are different from normal endothelial cells (NECs) in various aspects such as gene expression profiles [4], [5].

We have compared the characteristics of TECs and NECs, and found that TECs have several abnormalities such as upregulation of specific genes [6]–[9] and cytogenetic abnormalities [10], [11]. Furthermore, compared with NECs, TECs show more angiogenic phenotypes as well as high proliferative and migratory abilities [12]. We also found that the expression of stem cell markers such as Sca-1, CD90, and multidrug resistance 1 (MDR1) is upregulated in TECs compared with that in NECs. In addition, TECs form spheres and show a differentiation ability for osteoblasts [12]. These results suggest that stem-like cells exist in tumor blood vessels.

It has been reported that bone marrow-derived hematopoietic stem cells [13], [14] and resident endothelial stem/progenitor cells [15] play important roles in physiological angiogenesis during embryogenesis and pathological angiogenesis at the location of ischemia. However, the contribution of a stem cell population residing within blood vessels to tumor angiogenesis is still unclear.

Aldehyde dehydrogenase (ALDH) is an enzyme that plays a key role in the metabolism of aldehydes. Recent studies show that several stem cell types including hematopoietic stem cells [16] and neural stem cells [17] possess high ALDH activities. Thus, ALDH is used extensively as a stem cell marker.

In this study, we isolated ALDH^high^ and ALDH^low^ TECs, and compared their phenotypes to reveal the role of stem-like TECs in tumor angiogenesis.

## Materials and Methods

### Cell lines and culture conditions

Human microvascular endothelial cells (HMVECs) were obtained from Lonza (Tokyo, Japan) and cultured in endothelial cell growth medium (EGM-2MV; Lonza, Basel, Switzerland). A highly metastatic human melanoma cell line (A375SM) was a kind gift from Dr. Isaiah J Fidler (MD Anderson Cancer Center, Houston, TX, USA) [18]. A375SM cells were cultured in minimal essential medium (MEM; Gibco, Grand Island, NY, USA) supplemented with 10% fetal bovine serum (FBS) (10% MEM).

### Isolation of TECs and NECs

TECs were isolated from human melanoma xenografts in nude mice, and NECs were isolated from the dermis of the nude mice as controls according to a previous report [10]. All animal experimentation was approved by the Hokkaido University Ethics Committee (Permit No. 08–0296), and animal care was in accordance with the institutional guidelines of Hokkaido University. A375SM cells were injected subcutaneously into nude mice. The tumors were excised upon reaching a diameter of more than 10 mm. All surgery was performed under isoflurane anesthesia, and all efforts were made to minimize suffering. TECs and NECs were isolated using a magnetic-activated cell sorting system (Miltenyi Biotec, Auburn, CA, USA) with FITC-anti-CD31. CD31-positive cells were sorted and plated on fibronectin-coated culture plates in EGM-2MV containing 20% FBS. Diphtheria toxin (500 ng/mL; Calbiochem, San Diego, CA, USA) was added to TEC subcultures to eliminate any remaining human tumor cells and to NEC subcultures for technical consistency. A few weeks later, the subcultured TECs and NECs were subjected to a second purification round using FITC-BS1-B4.

The cells were cultured at 37°C in a humidified atmosphere with 5% CO_2_.

### RT-PCR and real-time RT-PCR

Total RNA was extracted from each type of endothelial cell using an RNeasy MicroKit (Qiagen, Valencia, CA, USA). First-strand cDNA was then synthesized with ReverTra-Plus (Toyobo Co., Osaka, Japan). Real-time RT-PCR was performed using SsoFast Evagreen Super mix (Bio-Rad, Hercules, CA, USA). Cycling conditions followed the manufacturer's instructions, and CFX Manager was used for analyses (Bio-Rad). Expression levels were normalized to glyceraldehyde-3-phosphate dehydrogenase (GAPDH) expression. The primers used were as follows.

mouse CD31: forward, 5′-TGCTCTCGAAGCCCAGTATT-3′; reverse, 5′-ATGGGTGCAGTTCCATTTTC-3′

mouse CD105: forward, 5′-CTTCCAAGGACAGCCAAGAG-3′; reverse, 5′-GGGTCATCCAGTGCTGCTAT-3′

mouse CD144: forward, 5′-CAGCACTTCAGGCAAAAACA-3′; reverse, 5′-TTCTGGTTTTCTGGCAGCTT-3′

mouse vascular endothelial growth factor receptor 1 (VEGFR1): forward, 5′-GAGGAGGATGAGGGTGTCTATAGGT-3′; reverse, 5′-GTGATCAGCTCCAGGTTTGACTT-3′

mouse vascular endothelial growth factor receptor 2 (VEGFR2): forward, 5′-GGCAAATGTGTCAGCTTTGTACA-3′; reverse, 5′-CAAAGCATTGCCCATTCGAT-3′

mouse CD11b: forward, 5′-GATGGGAAATGCAAAGAGGA-3′; reverse, 5′-AGGGTCTAAGCCAGGTCATAAG-3′

mouse CD45: forward, 5′-CCTCAAACTTCGACGGAGAG-3′; reverse, 5′-CACTTGCACCATCAGACACC-3′

human heparin-binding epidermal growth factor-like growth factor (HB-EGF): forward, 5′-CGGCCGGGACCGGAAA-3′; reverse, 5′-CCTGTTTGGTGTGG-3′

mouse GAPDH: forward, 5′-TCTGACGTGCCGCCTGGAG-3′; reverse, 5′-TCGCAGGAGACAACCTGGTC-3′

human GAPDH: forward, 5′-ACAGTCAGCCGCATCTTCTT-3′; reverse, 5′-GCCCAATACGACCAAATCC-3′

mouse VEGF-A: forward, 5′-GATTGAGACCCTGGTGGACATC-3′; reverse, 5′-CACACAGGAGGGCTTGAAGA-3′

mouse Sca-1: forward, 5′-GAAGAGGCAGAATTCCAAGG-3′; reverse, 5′-ATGTGGGAACATTGCAGGAC-3′

mouse CD90: forward, 5′-CTGGTGAACCAAAACCTTCG-3′; reverse, 5′-GCACGTGCTTCCTCTTCTCT-3′

mouse MDR1: forward, 5′-ATCCGGGAGCAGAAGTTTGA-3′; reverse, 5′-GCACCAAAGACAACAGCAGA-3′

mouse interleukin (IL)-6: forward, 5′-AGCTGGAGTCACAGAAGGAGTGGC-3′; reverse, 5′-GGCATAACGCACTAGGTTTGCCGAG-3′

mouse ALDH: forward, 5′-TCCGTCATGACCACCAGGTGCTTTCC-3′; reverse, 5′-ACAACACCTGGGGAACAGAGCAG-3′

mouse fibroblast growth factor-2 (FGF-2): forward, 5′- AGCGGCTCTACTGCAAGAAC-3′; reverse, 5′-TGGCACACACTCCCTTGATA-3′

human ALDH: forward, 5′-TGTTAGCTGATGCCGACTTG-3′; reverse, 5′-TTCTTAGCCCGCTCAACACT-3′.

### Cell proliferation assay

Cell proliferation was compared between TECs and NECs. After serum starvation for 16 h in Endothelial Basal Medium-2 (EBM-2; Lonza), 2×10^3^ cells per well were seeded in 96-well plates in EBM-2 with 0.5% FBS. Cell proliferation was measured every day for 3 days by an MTS [3-(4,5-dimethylthylthiazol-2-yl)-5-(3-carboxymethoxyphenyl)-2-(4-sulfophenyl)-2H-tetrazolium] assay (Promega, Madison, WI, USA).

Cell proliferation was also compared between ALDH^high^ and ALDH^low^ TECs. A total of 2.5×10^3^ cells per well were seeded in 96-well plates in EGM-2MV. Cell proliferation was measured every day for 3 days by the MTS assay.

### Cell migration assay

Cell migration was measured using the Boyden chamber method. In the upper chamber, 1.5×10^5^ cells were seeded in EBM-2 with 0.5% FBS. Vascular endothelial growth factor (VEGF; 10 ng/mL) was added to the lower chamber as a chemoattractant. After 4 h of incubation at 37°C, the cells that migrated through the fibronectin-coated polycarbonate filter (8-µm pores: Nuero Probe Inc., Gaithersburg, MD, USA) were fixed in 10% formaldehyde (Wako, Osaka, Japan) and stained with hematoxylin (Wako). The experiment was repeated three times with similar results.

### Flow cytometric analysis of ALDH activity

To analyze ALDH enzymatic activity and isolate the cell population with high ALDH activity, we used an ALDEFLUOR kit (StemCell Technologies, Durham, NC, USA) according to the manufacturer's instructions. Cells were suspended in ALDEFLUOR assay buffer containing ALDH substrate bodipy-aminoacetaldehyde (BAAA) and incubated for 40 min at 37°C. BAAA was taken up by live cells and converted into bodipy-aminoacetate by intracellular ALDH, which yields bright fluorescence. As a negative control, cells were stained under identical conditions with the specific ALDH inhibitor diethylaminobenzaldehyde. The highly ALDH-positive population (ALDH^high^) was detected using a FACS Aria II (BD Biosciences, San Jose, CA, USA) with a 488-nm blue laser and standard FITC 530/30-nm bandpass filter.

### Stemness spheroid assay

A cell suspension was seeded in a 96-well plate containing a micro sphere array chip (STEM Biomethod, Kitakyusyu, Japan), and 20 cells were seeded into microwells containing culture medium according to the manufacturer's instructions.

### Tube formation assay

Matrigel tube formation assays were performed to assess in vitro angiogenesis. Growth factor-reduced Matrigel (BD Biosciences) was added to each well of 24-well plates and incubated at 37°C for 30 min to allow the matrix solution to solidify. Cells were harvested and resuspended in EBM-2 containing 0.5% FBS and then seeded at a density of 1×10^5^ cells per well, followed by incubation at 37°C for 12 h. Tube formation was observed under an inverted microscope (CKX41; Olympus, Tokyo, Japan). Experimental results were recorded at three different times with similar results. The number of tube junctions was counted.

### Western blotting

Western blotting was performed using antibodies specific for Akt, phosphorylated Akt (p-Akt), β-actin, and a horseradish peroxidase-conjugated secondary antibody as described previously [19]. ALDH^high/low^ cells were treated with VEGF (50 ng/ml) for 30 min and then lysed as described previously [19].

### Human tissue samples

Human tissue samples were obtained from Hokkaido University Hospital. All protocols were approved by the Hokkaido University Ethics Committee, and written informed consent was obtained from each patient before surgery. Surgically resected tissues from patients diagnosed with renal cell carcinoma (RCC) were analyzed. The specimens included tumor tissues and corresponding normal renal tissues. A portion of the tissue samples was snap-frozen immediately in liquid nitrogen and stored at −80°C for immunohistochemistry. Final diagnosis of RCC was confirmed by pathological examination of formalin-fixed surgical specimens.

### Immunohistochemistry

Mouse tumor tissues were dissected from A375SM melanoma and HSC3 oral carcinoma xenografts in nude mice. Human tissue samples were obtained from excised RCC and normal kidney tissues of patients. Tumor specimens embedded in cryocompound (Tissue-Tek; Miles, Elkhart, IN, USA) were immediately immersed in liquid nitrogen and then cut into sections using a cryotome. The frozen sections were fixed in 4% paraformaldehyde for 10 min and then blocked with 2% goat and 5% sheep sera in PBS for 30 min. Mouse sections were double stained with a primary anti-ALDH1A1 antibody (Abcam, Cambridge, UK), Alexa 594-conjugated anti-rabbit IgG (Invitrogen, Tokyo, Japan), and Alexa 647-conjugated anti-mouse CD31 antibody (Biolegend, San Diego, CA, USA). Human sections were double stained with a primary anti-ALDH1A1 antibody (Abcam), Alexa 594-conjugated anti-rabbit IgG (Invitrogen), and Alexa 647-conjugated anti-human CD31 antibody (Biolegend). All immunostained samples were counterstained with DAPI (Roche Diagnostics, Mannheim, Germany) and visualized under a Fluo View FV1000 confocal microscope (Olympus).

### Preparation of conditioned medium (CM)

A375SM cells were seeded and cultured in 10% MEM until 70–80% confluence. Then, the culture medium was replaced with fresh medium. After 18–20 h of incubation, the culture supernatant was collected and passed through a 0.22-µm filter (Millipore, Billerica, MA, USA). HMVECs were exposed to fresh CM for 5 days with the CM changed after 2 days. For the control, HMVECs were incubated for 18–20 h in 10% MEM, and then the HMVEC CM was collected as described above.

### Statistical analysis

Differences between groups were evaluated using the Student's t-test. P<0.05 was considered significant, and p<0.01 was considered highly significant.

## Results

### Isolation and characterization of TECs and NECs

To compare the phenotypes of TECs and NECs, TECs were isolated from A375SM xenografts in nude mice and NECs were isolated from the dermis of normal nude mice as reported previously [10].

**Figure 1:**
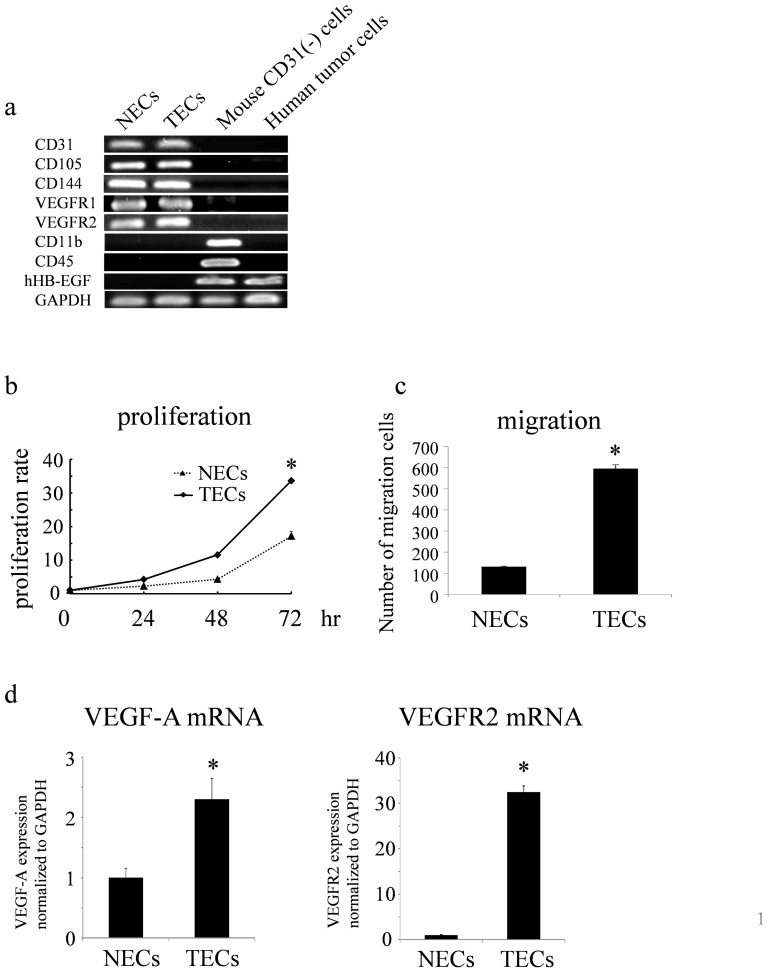
Isolation and characterization of TECs and NECs. a. Cultured TECs and NECs were positive for CD31, CD105, CD144, VEGFR1, and VEGFR2 as determined by RT-PCR. TECs and NECs were negative for the monocyte marker CD11b and hematopoietic marker CD45. Human HB-EGF expression was detected in human tumor cells but not in TECs or NECs. b. Cell proliferation was measured every day for 3 days by MTS assays (*p<0.05). c. Cell migration towards VEGF was analyzed using a Boyden chamber. Cells on the filter were fixed and stained with hematoxylin after 4 h of incubation. Cells that migrated to the underside of the filter were counted (*p<0.05). d. Relative expression of VEFG-A and VEGFR2 to GAPDH in TECs and NECs was measured using real-time RT-PCR (*p<0.01).

The expression of endothelial markers CD31, CD105, CD144, VEGFR1, and VEGFR2 in TECs and NECs was confirmed by RT-PCR. Isolated endothelial cells were negative for the monocyte marker CD11b and hematopoietic marker CD45. These results indicated that the isolated endothelial cells were highly pure. In addition, mRNA expression of human HB-EGF was not detected in mouse TECs, demonstrating that the TECs were not contaminated with human tumor cells (Fig. 1a).

Compared with NECs, it has been reported that TECs show a highly angiogenic phenotype [12], [20]. Cell proliferation was compared between TECs and NECs by MTS assays. The proliferation rate of TECs was significantly higher than that of NECs (Fig. 1b). Next, cell migration towards VEGF was analyzed using a Boyden chamber. We found that the migration of TECs migrated was faster than that of NECs (Fig. 1c).

To analyze and compare the expression of angiogenesis-related genes in TECs and NECs, the expression of VEGF-A and its receptor, VEGFR2, was detected by real-time PCR. Compared with NECs, the mRNA expression level of VEGF-A was 2.3-fold higher and that of VEGFR2 was 32-fold higher in TECs (Fig. 1d). These results indicated that TECs had a more pro-angiogenic phenotype than that of NECs, which was consistent with our previous studies [12], [20].

### TECs exhibit a stem-like phenotype

We have previously reported that TECs exhibit stem cell characteristics [12]. Therefore, we investigated the stem cell characteristics of the isolated endothelial cells. Previous studies have reported that TECs can transdifferentiate into alkaline phosphatase-positive cells. [21]. We also found that TECs exhibit alkaline phosphatase activity after 3 days of culture in osteogenic differentiation medium [12].

**Figure 2:**
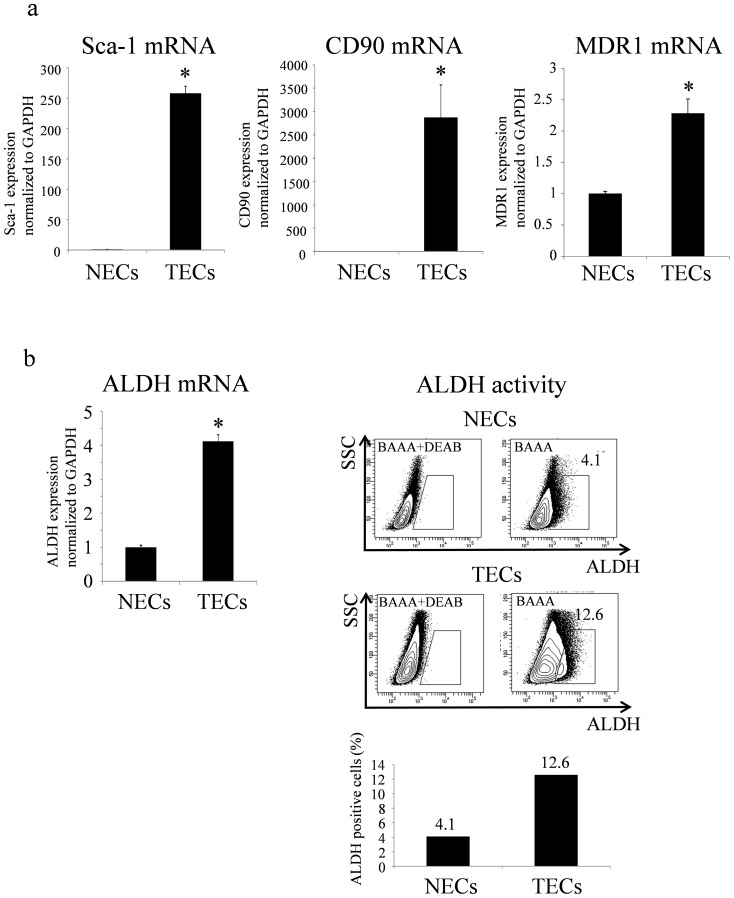
TECs exhibit a stem like phenotype. a. Relative expression of Sca-1, CD90, and MDR1 to GAPDH in TECs and NECs was measured using real-time RT-PCR (*p<0.01). b. ALDH mRNA expression was upregulated in TECs as determined by real-time RT-PCR (*p<0.01). ALDH activity was higher in TECs than that in NECs based on analysis of ALDH activity by flow cytometry.

Compared with NECs, these findings demonstrate that TECs include a larger population of stem-like cells. Real-time PCR revealed upregulation of stem cell markers including Sca-1, CD90, and MDR1 in TECs compared with that in NECs (Fig. 2a).

ALDH is a stem cell marker that is used extensively as a marker of hematopoietic stem cells [16] and neural stem cells [17]. Furthermore, recent studies have identified ALDH enzymatic activity as a potential marker for cancer stem cells [22]. ALDH mRNA expression in TECs was 4-fold higher than that in NECs (Fig. 2b). The ALDH activity of TECs was also higher than that of NECs in ALDH activity assays. A representative analysis showed that 12.6% of TECs were ALDH^high^ cells, whereas only 4.1% of NECs were ALDH^high^ cells (Fig. 2b).

### Isolation of ALDH^high^ and ALDH^low^ TECs

**Figure 3:**
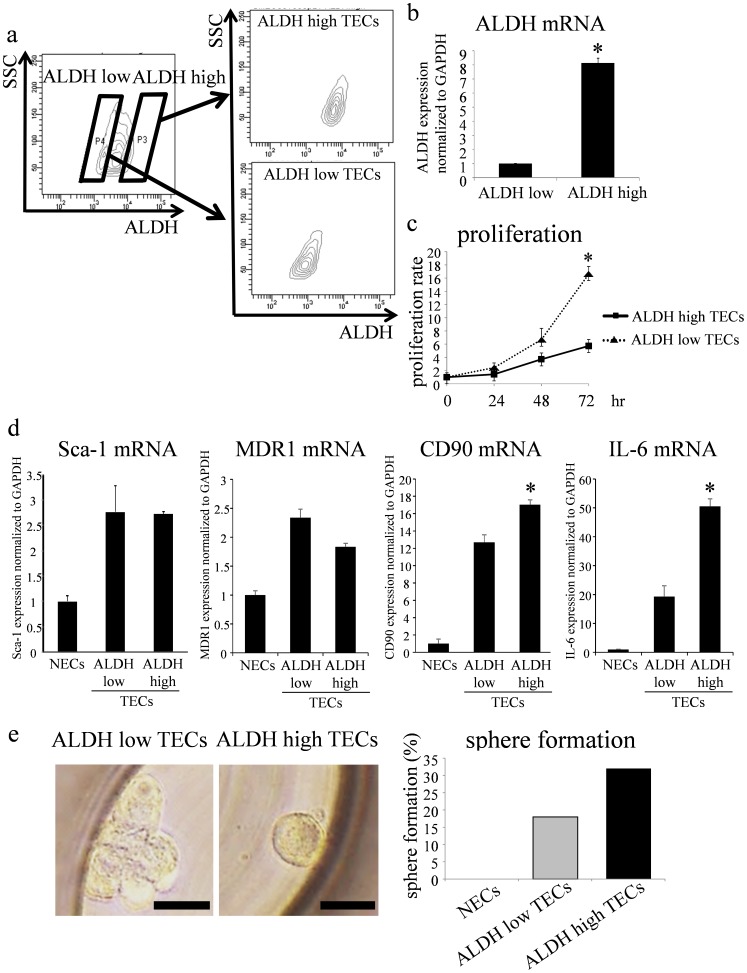
Isolation of ALDH^high^ cells by FACS. a. TECs with high or low ALDH activity (ALDH^high^ and ALDH^low^ TECs, respectively) were isolated by FACS. b. Analysis of ALDH mRNA expression by real-time RT-PCR confirmed that the sorted ALDH^high/low^ TECs were highly pure (*p<0.01). c. Cell proliferation was measured every day for 3 days by MTS assays. The proliferation of ALDH^high^ TECs was significantly slower than that of ALDH^low^ TECs (*p<0.01). d. Relative expression of Sca-1, CD90, MDR1, and IL-6 to GAPDH in ALDH^high/low^ TECs was measured using real-time RT-PCR (*p<0.01). e. ALDH^high^ TECs showed spheroid morphological features with a smooth surface and high circularity at 5 days after seeding into microwells in the stemness spheroid assay. ALDH^high^ TECs formed spheres at a higher frequency than that of ALDH^low^ TECs. Scale bar: 30 µm.

Previous reports have described the mobilization of bone marrow-derived circulating endothelial progenitor cells [13], [14] and the role of resident endothelial stem cells [15] in angiogenesis. However, the roles of stem cells residing within tumor blood vessels in cancer biology are still unclear. To characterize the phenotype of stem-like TECs, ALDH^high^ and ALDH^low^ TECs were sorted according to their ALDH activity by fluorescence-activated cell sorting (FACS) (Fig. 3a). ALDH mRNA expression was 8-fold higher in ALDH^high^ TECs than that in ALDH^low^ TECs, suggesting that the sorted ALDH^high/low^ TECs were highly pure (Fig. 3b). ALDH^high^ TEC proliferation was slower than that of ALDH^low^ TECs (Fig. 3c), suggesting that ALDH^high^ TECs resemble dormant cells. We compared the expression levels of some stem cell markers in ALDH^high^ and ALDH^low^ TECs by real-time PCR. The mRNA expression levels of Sca-1, MDR1, CD90, and IL-6 were higher in ALDH^high/low^ TECs than those in NECs. There was no difference in the mRNA expression of Sca-1 and MDR1 in ALDH^high^ and ALDH^low^ TECs. However, CD90 mRNA expression was 1.3-fold higher in ALDH^high^ TECs than that in ALDH^low^ TECs. Furthermore, the expression level of IL-6 mRNA was 2.6-fold higher in ALDH^high^ TECs than that in ALDH^low^ TECs (Fig. 3d).

Next, we compared the sphere formation abilities of ALDH^high^ and ALDH^low^ TECs. ALDH^high^ TECs formed spheres at a higher frequency than that of ALDH^low^ TECs (Fig. 3e). These results suggest that ALDH^high^ TECs may have more stem cell characteristics than ALDH^low^ TECs.

### ALDH^high^ TECs show a highly angiogenic phenotype

**Figure 4:**
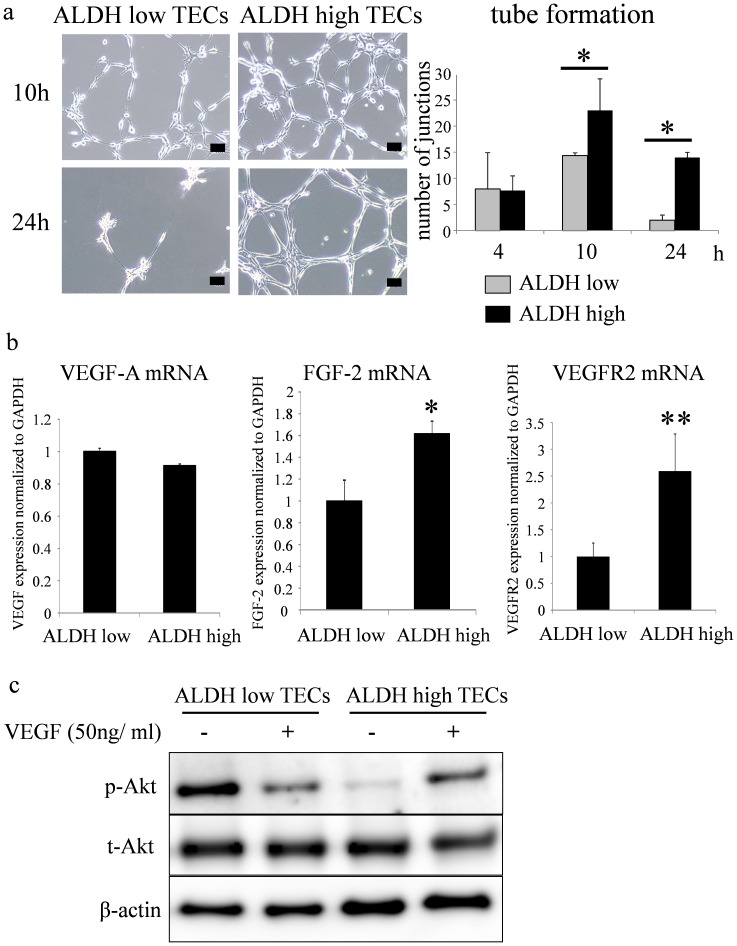
ALDH^high^ TECs show a highly angiogenic phenotype. a. The number of tube junctions (arrows) was counted at 10 and 24 h after seeding onto Matrigel in a tube formation assay in vitro (*p<0.05). Scale bar: 50 µm. b. Relative expression of VEGF-A, FGF-2, and VEGFR2 to GAPDH in ALDH^high/low^ TECs was measured using real-time RT-PCR (*p<0.05; **p<0.01). c. Levels of p-Akt were determined by western blotting using an anti-p-Akt antibody. The membrane was stripped and re-incubated with anti-total Akt (t-Akt) and -β-actin antibodies. After treatment with VEGF for 20 min, Akt was more activated in ALDH^high^ TECs than that in ALDH^low^ TECs.

To analyze the angiogenic phenotypes of ALDH^high^ TECs, we performed in vitro tube formation assays. After the endothelial cells were seeded onto Matrigel in a very low concentration of serum (0.5% FBS), the number of tube junctions was counted after 10 and 24 h of incubation. As a result, we observed a significantly higher number of tube junctions formed by ALDH^high^ TECs than that formed by ALDH^low^ TECs (Fig. 4a). Furthermore, the tubular networks formed by ALDH^high^ TEC were sustained after 24 h of incubation, whereas ALDH^low^ TECs could not sustain their tube formation. These results suggest that ALDH^high^ TECs, but not ALDH^low^ TECs, contribute to angiogenesis even under nutrition-exhausted conditions.

### Angiogenesis-related genes are upregulated in ALDH^high^ TECs

Our previous report showed upregulation of angiogenesis-related genes such as VEGF-A in TECs, which might affect angiogenesis in an autocrine manner [19]. To determine the mechanism of the highly angiogenic phenotypes of ALDH^high^ TECs, the expression levels of angiogenesis-related genes were compared in ALDH^high^ and ALDH^low^ TECs by real-time PCR. There was no difference in the expression of VEGF-A in ALDH^high/low^ TECs (Fig. 4b). However, FGF-2 mRNA expression was 1.6-fold higher in ALDH^high^ TECs than that in ALDH^low^ TECs. Furthermore, the expression level of VEGFR2 mRNA was 2.6-fold higher in ALDH^high^ TECs than that in ALDH^low^ TECs (Fig. 4b). These results suggested that ALDH^high^ TECs were more sensitive to VEGF-A through upregulation of its receptor, VEGFR2. Because both ALDH^high^ and ALDH^low^ TECs express VEGF, VEGFR2 upregulation may be one of the mechanisms underlying the highly angiogenic property of ALDH^high^ TECs. In fact, Akt was highly activated by VEGF stimulation in ALDH^high^ TECs compared with that in ALDH^low^ TECs (Fig. 4c). These results suggested that the higher level of VEGFR2 expression may be at least one of the reasons why Akt was more activated by VEGF stimulation in ALDH^high^ TECs compared with that in ALDH^low^ TECs.

### ALDH is expressed in mouse tumor blood vessels in vivo

To analyze in vivo ALDH expression in TECs, double immunofluorescence staining of A375SM tumor and oral carcinoma xenografts in mice was performed using anti-ALDH and anti-CD31 antibodies.

**Figure 5:**
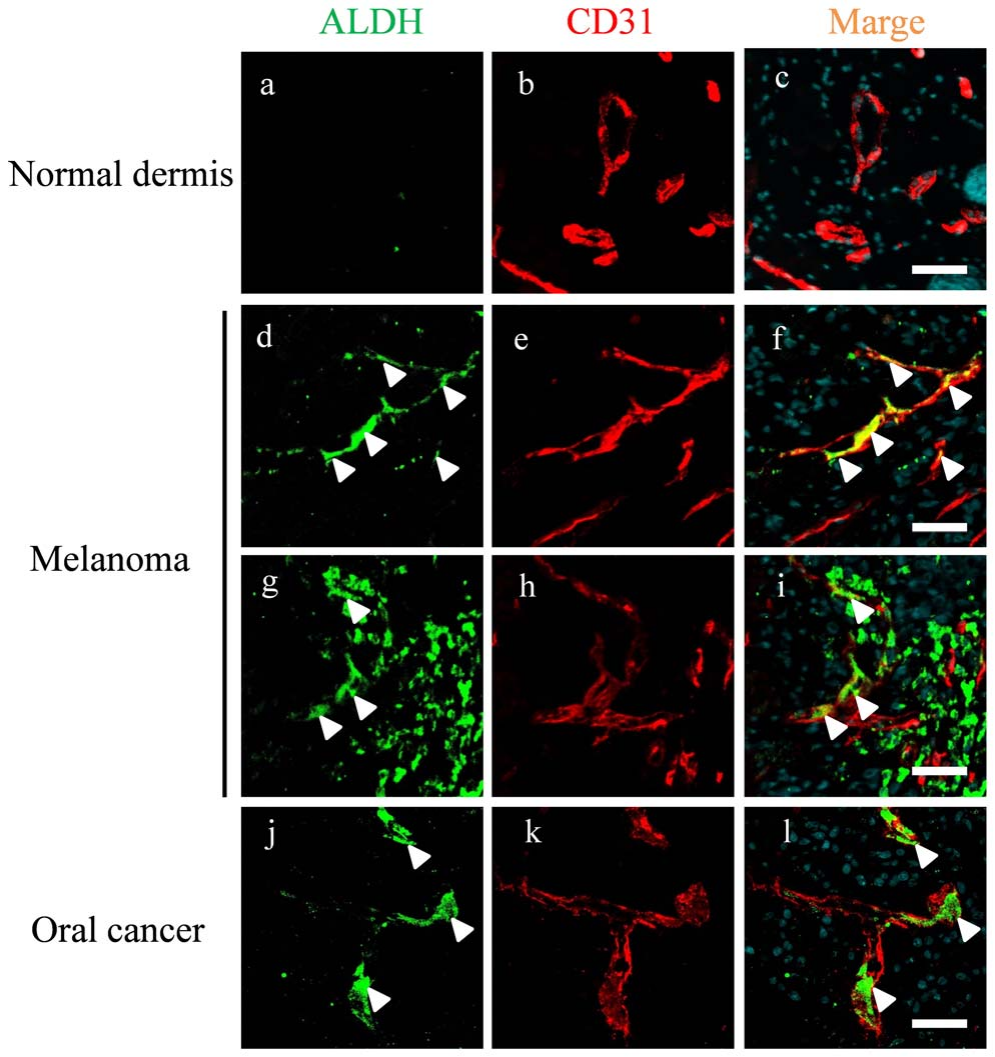
ALDH is expressed in mouse tumor blood vessels in vivo. Double immunofluorescence staining for endothelial markers CD31 and ALDH in normal mouse tissue (dermis) (a–c), A375SM melanoma xenografts () (d–i), and oral carcinoma xenografts (j–l). Merged images (white arrow) show co-localization of ALDH (green) and CD31 (red) in situ. Nuclei were counterstained with DAPI. Scale bar: 40 µm.

ALDH was hardly expressed in normal blood vessels in vivo (Fig. 5a–c); however, ALDH was expressed in the tumor blood vessels of melanoma (Fig. 5d–i) and oral carcinoma xenografts (Fig. 5j–l). These results suggest that the blood vessels of some types of cancers contain ALDH^high^ endothelial cells. Furthermore, the ALDH expression pattern was heterogeneous in tumor blood vessels, suggesting that stem-like endothelial cells exist in tumor blood vessels in vivo.

### ALDH is expressed in human tumor blood vessels

**Figure 6:**
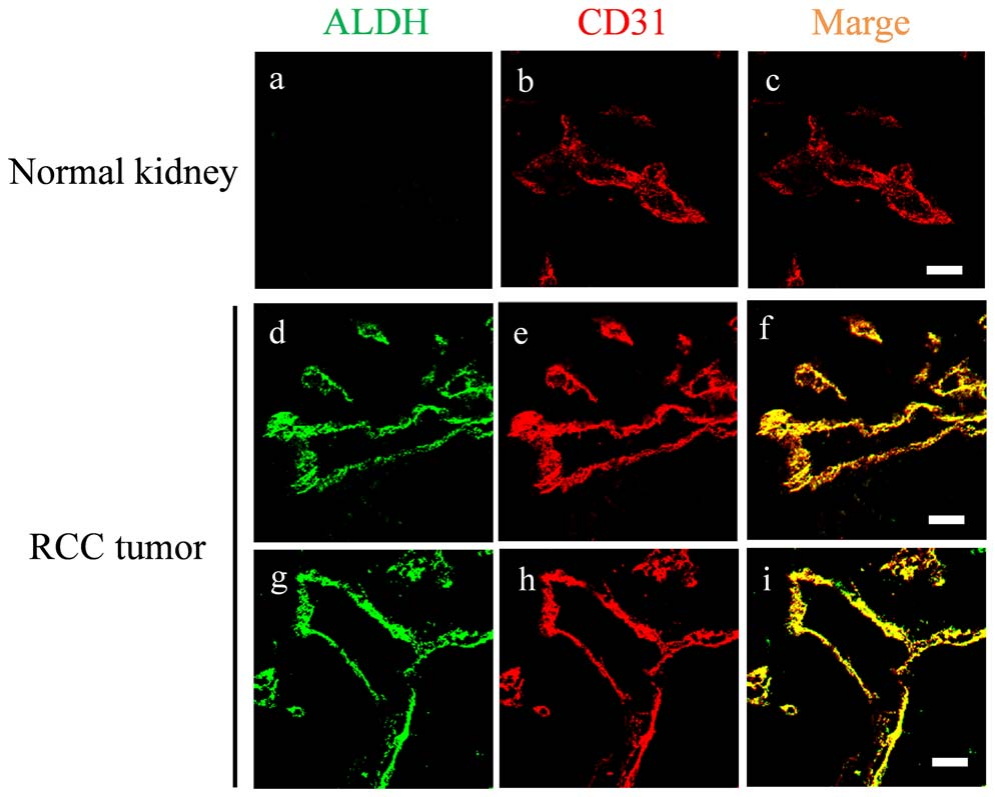
ALDH is expressed in human tumor blood vessels. Double immunofluorescence staining for endothelial markers CD31 and ALDH in normal human kidney tissue (a–c) and RCC tumor tissue (d–i). Merged images show co-localization of ALDH (green) and CD31 (red) in situ. Scale bar: 40 µm.

To analyze whether ALDH is expressed in human tumor blood vessels as well as in mouse tumor blood vessels, we performed double immunofluorescence staining of the frozen sections of human renal tumors and normal kidney tissues using anti-ALDH and anti-CD31 antibodies. Because RCC is known to be angiogenic, we chose RCC sections as for immunohistochemistry. ALDH staining was negative in normal blood vessels (Fig. 6a–c), but was strongly positive in tumor blood vessels (Fig. 6d–i). These results suggest that ALDH was upregulated in hTECs in vivo and may be involved in tumor angiogenesis in cancer patients.

## Discussion

Recently, the presence of stem-like endothelial cells has been suggested in pre-existing blood vessels [15]. We have reported that TECs show upregulation of some stem cell markers, and can differentiate into cells forming bone-like tissue [12]. However, there are no reports on the functions of stem-like TECs. In this study, we demonstrated that there are stem-like TECs in tumor blood vessels. TECs had high expression of stem cell markers Sca-1, CD90, and MDR1, suggesting that they possess some stem cell characteristics [12]. In addition, TECs showed high ALDH enzymatic activity that has been also used as a hallmark of stem cells. Previous reports demonstrate that ALDH may identify cell populations enriched with hematopoietic stem cells [16], neural stem cells [17], and cancer stem cells [22]. Thus, we isolated ALDH^high/low^ TECs and compared their phenotypes.

There have been several reports on the heterogeneity of the tumor endothelium [12], [23]. In our study, stem-like TECs expressing ALDH were sparsely distributed in tumor blood vessels, which supported endothelial cell heterogeneity.

Tumor angiogenesis is regulated by a balance of stimulators (e.g., VEGF-A, hepatocyte growth factor, and FGF-2) and inhibitors (e.g., endostatin and vasohibin). Among these factors, the VEGF-A/VEGFR2 signaling pathway is the most potent inducer. In the tumor microenvironment, both tumor and stromal VEGF contribute to angiogenesis [24]. In this study, we observed that ALDH^high^ TECs, but not ALDH^low^ TECs, formed tubes on Matrigel even without growth factors. Furthermore, compared with ALDH^low^ TECs, ALDH^high^ TECs sustained their tube formation for a longer period. In addition, VEGFR2 mRNA expression was higher in ALDH^high^ TECs compared with that in ALDH^low^ TECs, suggesting that activation of the VEGF-A/VEGFR2 signaling pathway is one of the mechanisms underlying the highly angiogenic property of ALDH^high^ TECs.

Although there are increasing studies of TEC abnormalities, the mechanisms of these abnormalities are still unclear. We previously found that VEGF-A secreted from tumor cells upregulates MDR1 mRNA expression in NECs [25]. Thus, we speculated that pre-existing endothelial cells in tumor vessels acquire a stem cell phenotype through the effects of tumor-derived factors such as VEGF. To determine the regulatory mechanism of ALDH expression in TECs, we analyzed the effect of tumor-derived factors on NECs using tumor CM. Compared with control CM-treated HMVECs, ALDH mRNA expression levels were increased by 3.6-fold in HMVECs exposed to tumor CM (Fig. S1). These results suggested that tumor-derived factors may be involved in the upregulation of ALDH in TECs. However, further study is needed to reveal the detailed mechanism by which TECs acquire a stem cell phenotype in the tumor microenvironment.

In summary, we have documented the existence of stem-like TECs that highly express ALDH and show a pro-angiogenic phenotype. Stem-like TECs may have an essential role in tumor angiogenesis, and therefore contribute to tumor progression. Targeting stem-like TECs would be an attractive strategy for anti-angiogenic therapy.

## Supporting Information

Figure S1 **Tumor CM up-regulates ALDH mRNA expression in HMVECs.** HMVECs were cultured for 5 days in CM, and then their ALDH mRNA expression level was measured using real-time RT-PCR (*p<0.01). After tumor CM treatment, ALDH mRNA expression in HMVECs was significantly upregulated compared with that in the control.
(TIF)
