# Supplementary figures and images for: Identification of Tumor Endothelial Cells with High Aldehyde Dehydrogenase Activity and a Highly Angiogenic Phenotype
Source: PLoS One. 2014 Dec 1;9(12):e113910. doi: 10.1371/journal.pone.0113910 (PMC4250080; doi:10.1371/journal.pone.0113910)

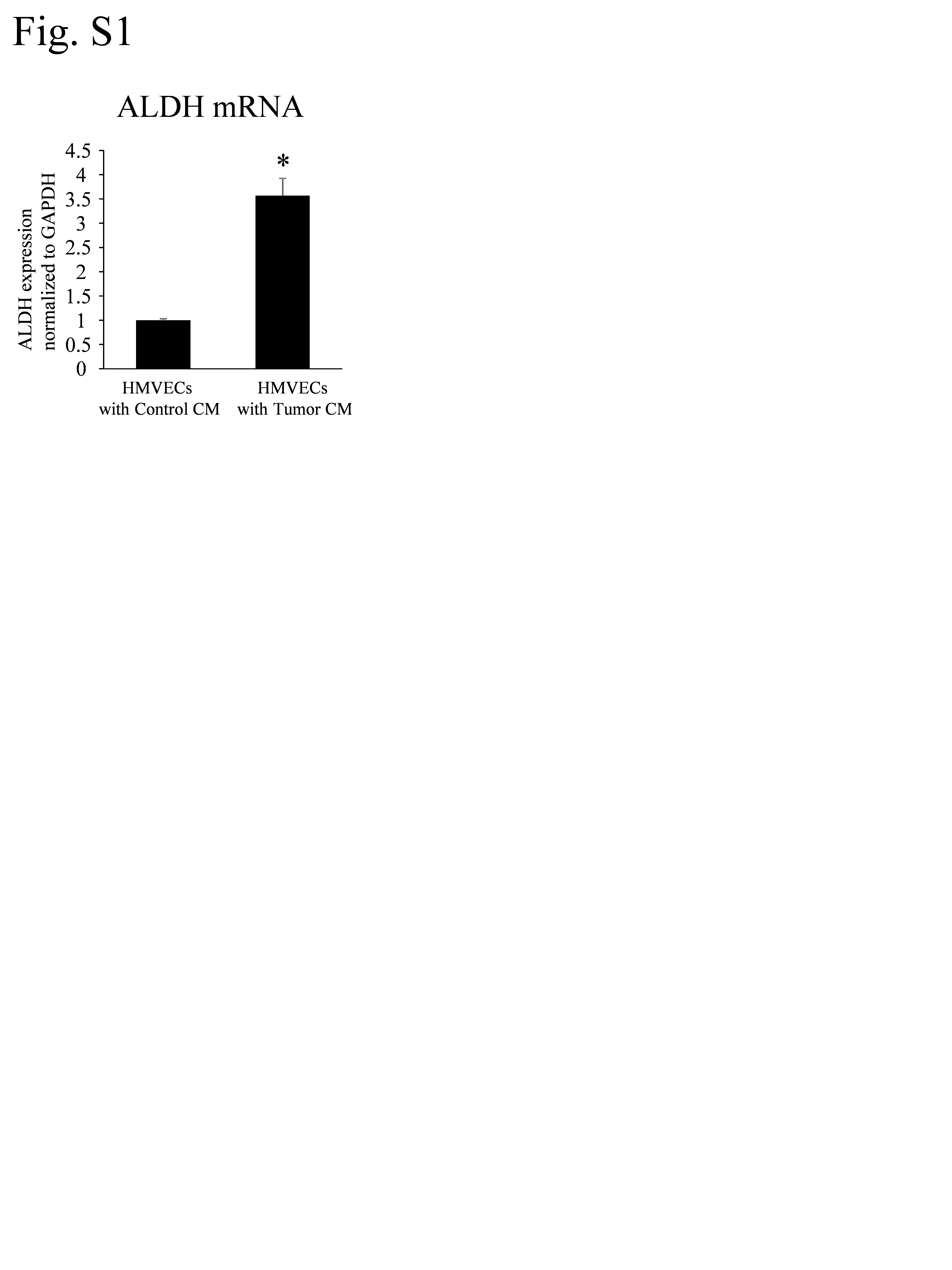

Supplement: Figure S1 — Tumor CM up-regulates ALDH mRNA expression in HMVECs. HMVECs were cultured for 5 days in CM, and then their ALDH mRNA expression level was measured using real-time RT-PCR (*p<0.01). After tumor CM treatment, ALDH mRNA expression in HMVECs was significantly upregulated compared with that in the control. (TIF) [file pone.0113910.s001.tif]
